# Good or Bad? The Ambivalent Leader-Follower Relationships

**DOI:** 10.3389/fpsyg.2021.690074

**Published:** 2021-08-09

**Authors:** Qinglin Zhao, Wenxia Zhou

**Affiliations:** School of Labor and Human Resources, Renmin University of China, Beijing, China

**Keywords:** ambivalent relationships, paradox view, conservation of resources theory, workplace relationships, stressor

## Abstract

Researchers have emphasized the positive and negative influences of ambivalent leader-follower relationships, but it is not clear when the ambivalent relationship is associated with good or bad influences. To answer this question, we reviewed the definition and identified 10 different types of ambivalent leader-follower relationships. Further, we demonstrate that the negative outcomes (more inflexibility, disengagement, and worse performance) can be explained by the workplace stressor perspective, and that the positive outcomes (more flexibility, engagement, and better performance) can be explained by paradox view. Finally, drawing from conservation of resources (COR) theory, we integrate workplace stressor framework and the paradox view to address when the ambivalent leader-follower relationship is beneficial or detrimental for followers. We proposed that the degree of ambivalence, support from the third party, and integrative complexity of follower will influence the possible positive or negative influences. Limitations and future directions were also discussed.

## Introduction

The research on ambivalence has attracted attention from multiple fields, such as psychology, management, sociology, and marketing. Rothman et al. (2017) reported that 245 ambivalence-related studies have been published in A-level journals since 2000. At the interpersonal level, ambivalent relationships with the leader have attracted much attention (Owens et al., 2015; Zhang et al., 2015). It is not surprising given that leaders demonstrate different leadership styles at varying frequencies (Kelloway et al., 2006), the importance of effective interaction between the leader and follower in finishing job tasks (Nahum-Shani et al., 2014). Besides, the rapidly changing business environment requires leaders to be more “paradox-savvy” (Waldman and Bowen, 2016).

Previous research demonstrated that ambivalent leader–follower relationships may bring positive influences, negative influences, or different influences for different individuals or in different contextual conditions (Nahum-Shani et al., 2014). Due to the dual nature of ambivalences, it is of theoretical and practical implications to examine the boundary conditions that influence the outcomes. Albeit this importance, surprisingly, only few empirical studies, to the best knowledge of the authors, discussed the moderators between leader–follower ambivalent relationships and outcomes (De Cremer, 2003; Nahum-Shani et al., 2014; Lee et al., 2019; Suurd Ralph, 2019). In these articles, most of the argument is based on stress-buffering effects, which is narrow to understand these influences. Many scholars have called for more effort to elucidate other possible moderators (Kuwabara et al., 2010; Methot et al., 2017).

To further understand these contradictory findings of ambivalent leader-follower relationships in the workplace, we begin by reviewing the definition and types of ambivalent leader-follower relationships. We then summarize the outcomes, theoretical explanations, and empirical support for a generalized framework about how leader-follower ambivalent relationships may lead to positive and negative influences. Finally, we propose three moderators that may influence the positive or negative outcomes by applying the COR theory to integrate the present theoretical lens.

We contribute the ambivalent leader-follower relationship literature in the following ways. First, we applied the COR theory to explain why and when some ambivalent leader-follower relationships may lead to positive and negative outcomes; and three boundary conditions, namely, the extent of ambivalence, support from the third party, and integrative complexity of follower may moderate these outcomes. More importantly, we believe these three moderators work not only by buffering stress but also helpful in transferring stress to potential resources, which broaden our understanding. Second, we summarized the outcomes based on the flexibility-engagement outcome dimensions, and most findings are aligned with ambivalent literature (Rothman et al., 2017). Besides, we classified different ambivalent leader-follower relationships based on the typology of social network ties and found the most positive outcomes are from the “seems conflicting while compatible” ambivalent leader behaviors.

## What Is Ambivalent Leader-Follower Relationships

### Definition of Ambivalent Leader-Follower Relationships

To clarify what is included in the ambivalent leader-follower relationship, we first review “ambivalent relationship” related constructs (e.g., ambivalence, dissonance tie). Most definitions of ambivalence emphasized the simultaneously “positive and negative” orientations toward an object (Ashforth et al., 2014; Brennecke, 2020). Rothman et al. (2017) modified this conceptualization using “opposing orientation,” which explained that ambivalence may arise from simultaneously positive or negative emotions.

When ambivalence occurs between leader and follower, we call it “ambivalent leader–follower relationship.” Some conceptual and empirical studies have accumulated in the ambivalent leader-follower relationships, such as paradoxical leadership (Zhang et al., 2015), emotional complexity of leader (Rothman, 2017), leader inconsistency (De Cremer, 2003; Mullen et al., 2011, 2018), and leader hypocrisy (Greenbaum et al., 2015). This study mainly focuses on the outcomes or some specific ambivalent leadership behavior, and less attention was paid to the clarification of the ambivalent leader-follower relationship. We list some ambivalent leader-follower relationship definitions in Table 1. As we can see, these definitions mainly focus on one specific dimension. According to present empirical research, ambivalence may come from different interactions/behavior (e.g., leader hypocrisy), cognition (e.g., leader inconsistency in decision making), and emotions (e.g., emotional complexity), which is ignored by present definition.

**Table 1:** Key definitions about ambivalent relationship and leader–follower ambivalent relationship.

**Construct**	**Source**	**Definition**
Ambivalence	Rothman et al. (2017)	Simultaneous experience of opposing orientations toward an object or target.
Ambivalent tie	Ashforth et al. (2014)	Simultaneously positive and negative orientations toward an object.
Dissonant tie	Brennecke (2020)	Positive-negative multiplexity characterized by an individual's conflicting cognitions of another person.
Ambivalent leadership	Herr et al. (2019)	Simultaneously in positive and negative interaction with leaders.
Inconsistent leadership	Mullen et al. (2011)	Leaders are seen as being both transformational and passive.
Paradoxical leader	Zhang et al. (2015)	(1) combining self-centeredness with other-centeredness; (2) maintaining both distance and closeness; (3) treating subordinates uniformly, while allowing individualization; (4) enforcing work requirements, while allowing flexibility; and (5) maintaining decision control, while allowing autonomy.
Leader hypocrisy	Brunsson (1989)	Leader's word-deed misalignment.

Building on the refined definition of ambivalence (Rothman et al., 2017) and present leader-follower relationship research, we define the leader-follower ambivalent relationship as a simultaneous experience of opposing orientations toward leaders, the opposing orientation may come from opposing affection, cognition, or interaction (behavior).

### Type of Ambivalent Leader-Follower Relationships

As the definition of ambivalent leader-follower relationship indicates, the ambivalence may come from different sources. Previous literature indicated that the ambivalent can be classified into attitudinal ambivalence, emotional ambivalence (or mixed emotions), relational ambivalence, trait ambivalence, and expressed ambivalence (see Rothman et al., 2017, for a review). This classification fails to capture all kinds of ambivalence. For example, the ambivalent may come from opposing “expressed behavior” and “relational,” such as abusive supervisory from high LMX leaders. To solve this problem, drawing on a previous study about the type of relationship (Borgatti et al., 2009; Yang et al., 2019) and integrating existing leadership research, we argue that leader–follower relationship ambivalence may come from affect, cognition, complex tie^1^, behavior, and cross-ambivalence of this four. For example, when you were abused (behavior) by the leader with higher LMX (complex tie), it will also cause ambivalence. Thus, we have 10 types of leader-follower ambivalence relationships, and we list all the types and present research in Table 2.

**Table 2:** Ten types of ambivalent leader-follower relationships.

	**Affect-based**	**Cognition-based**	**Multiplex tie**	**Behavior**
Affect-based	***(1)*** Emotional complexity (Rothman, 2017)	——	——	——
Cognition-based	***(2)*** Incompetent & warmth (Suurd Ralph, 2019)	***(3)*** *Inconsistency in decision making (De Cremer, 2003)*	——	——
Multiplex tie	***(4)***	***(5)***	***(6)*** LMX ambivalence (Lee et al., 2019); Relational ambivalence (Guarana and Hernandez, 2015; Ingram, 2015)	——
Behavior	***(7)***	***(8)***	***(9)*** High LMX & abusive supervision (Lian et al., 2012)	***(10)*** Undermining & support (Duffy, 2002; Nahum-Shani et al., 2014); Safety-specific transformational leadership & passive leadership (Mullen et al., 2011); Paradoxical leadership behavior (Zhang et al., 2015; Jia et al., 2018; Shao et al., 2019; Fürstenberg et al., 2021); Leader humility and narcissism (Owens et al., 2015; Zhang et al., 2017); Leader hypocrisy (Greenbaum et al., 2015); Transformational leadership & supervisor incivility (Mullen et al., 2018); Transformational leadership & abusive supervision (Suurd Ralph, 2019); Supportive & Burdening (Herr et al., 2019); Visionary & empowering leadership (Kearney et al., 2019)

*Italic bold number in brackets is the possible types of leader–follower relationship ambivalence*.

To search all possible leader-follower ambivalence relationships, we used several keywords in topics to conduct the research on Web of Science, and these keywords were ambivalence^*^ AND leader^*^, inconsistency^*^ AND leader^*^, paradox^*^ AND leader^*^. We only focus on studies on the management field. Finally, 524 studies were identified. We read the abstract of all the studies and keep 12 related ones that mainly focus on the influence of ambivalent relationships. Considering the broad content of leadership, we also added another eight studies that include ambivalent leader-follower relationships. Finally, 20 studies were retained. We reviewed these 20 studies on leader-follower ambivalent relationships and found that current studies have covered six of the 10 possible types: ambivalent affect (emotional complexity), ambivalent cognition (leader inconsistency in decision making), ambivalent complex tie (LMX ambivalence), ambivalent leader behavior (support and undermine), ambivalent affect and cognition (incompetent and warmth), and ambivalent multiplex tie and behavior (abusive supervision from high LMX leader). About half of the research focuses on ambivalent leader behaviors, such as leader undermining and support (Duffy, 2002), paradoxical leadership behavior (Zhang et al., 2015), leader humility and narcissism (Owens et al., 2015), and visionary and empowering leadership (Kearney et al., 2019).

## Consequences of Ambivalent Leader–Follower Relationships

We now turn to a discussion of the impacts of ambivalent leader-follower relationships for followers in organizations. The effects of the ambivalent relationship can be organized into two key dimensions: flexibility and engagement (Rothman et al., 2017). Flexibility can be classified into cognitive flexibility, behavior flexibility, and emotional and physical flexibility. Engagement means the attitude or behavior toward the ambivalence, which includes disengagement (e.g., moving away) or engagement (e.g., moving toward). Most leader-follower ambivalent relationship outcomes can be classified into these two dimensions, and only few outcomes are new. Here, we name them as “performance and other outcomes” (such as job performance, goal clarity, and job engagement).

Leader-follower ambivalent relationships may bring positive or negative outcomes. Positive or negative affect is likely to play a critical role in deciding the positive or negative part (Rothman et al., 2017). We agree with the importance of affect, but other factors play an important role in influencing the outcomes. For example, ambivalence may bring some objective resources (e.g., new information or perspective). Considering these characteristics, we applied the COR theory to explain the outcomes, because COR covers the process of resource losses and gains, and it also emphasizes the “objective and culturally construed nature of the environment” rather than personal construal of individual (Hobfoll, 2001). Thus, here we argued that ambivalent is “threat of resources loss” or “opportunity to gain resources” that decides positive or negative outcomes.

On one hand, ambivalence can be regarded as a threat of resource loss, which leads to negative outcomes. First, ambivalent relationships trigger the feeling of stress (Nahum-Shani et al., 2014; Herr et al., 2019), loss of control, elicit negative affective responses (Lee et al., 2019), and even lead to negative physicals, such as somatic complaint (Duffy, 2002). Second, ambivalence makes followers feel uncertainty about themselves, which leads them to feel lower self-social esteem (De Cremer, 2003) or lower self-efficacy (Duffy, 2002). Third, these ambivalent in interaction make follower hard to interpret, and influence leaders' behavior (Suurd Ralph, 2019), to avoid this unpleasant and unpredictable experience and further resources loss, followers may choose to avoid the leaders or trigger the desire to replace leader (De Cremer, 2003; Suurd Ralph, 2019). Finally, these inflexibilities and disengagements lead to worse performance.

On the other, ambivalence can be regarded as opportunities to acquire resources and get positive outcomes by providing more information, guidance, and new perspectives. First, ambivalence means more different information, opinions, or guidance, which is beneficial to get positive outcomes. For example, paradoxical leadership gives individuals more guidance about what to do, leading individuals to have higher flexibility (Zhang et al., 2015). Second, based on this new information and perspectives, followers are more likely to show a proactive behavior. Third, followers may have positive opinions about leaders after gaining more cognitive and behavioral flexibility, which is helpful to form better leader-follower relationships. For example, leader humility and narcissism make follower think the leader is more effectiveness (Owens et al., 2015), may further lead to better engage in leader relationship. Finally, all new information, guidance, and performance.

In sum, the findings are aligned with previous findings, that is, ambivalent relationship may bring flexibility or inflexibility, and trigger engagement or disengagement (Rothman et al., 2017). In addition to these influences, leader-follower ambivalent relationships also influence work-related behavior and outcomes of followers, such as adaptative behavior, proactive behavior, job engagement, and performance. Of note, though most negative outcomes can be explained by the workplace stressor framework, other theoretical lens/mechanisms, such as procedural justice, were also mentioned (De Cremer, 2003). In this study, we mainly focus on the workplace stressor perspective.

## Good or Bad, What Moderators Influence Follower's Outcome?

**Figure 1:**
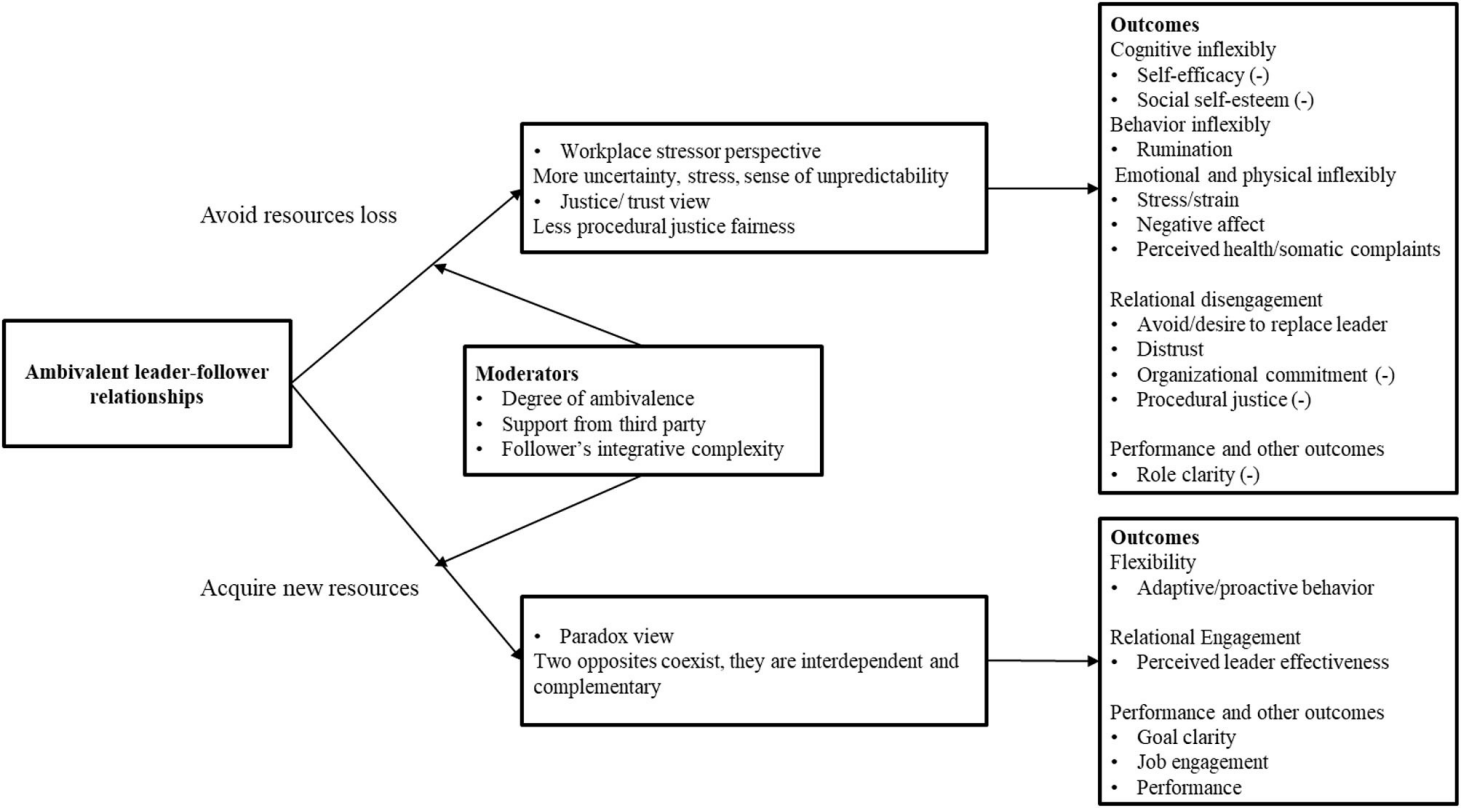
Ambivalent leader–follower relationships at work.

From the workplace stressor framework, ambivalence is the source of uncertainty, sense of unpredictability, which further brings negative outcomes; while from paradox view, ambivalence provides positive outcomes because of the integration of new perspectives and resources. We can interpret ambivalence from both perspectives simultaneously. This generates a question concerning what condition the leader-follower ambivalence is more likely to bring positive outcomes? Current research is limited in answering this question. To the knowledge of the authors, only gender (Suurd Ralph, 2019), self-esteem (De Cremer, 2003; Nahum-Shani et al., 2014), perceived organizational support (Lee et al., 2019), and perceived quality of work life (Nahum-Shani et al., 2014) were discussed, and all of these moderators were regarded as exerting their influence by stress-buffering effects^2^. Here, we argued that the ambivalence characteristics (degree of ambivalence), contextual characteristics (support from the third party), and individual differences (integrative thinking of follower) will moderate the relationship between leader–follower ambivalence and the outcomes. The model is presented in Figure 1.

### The Degree of Ambivalence

We propose that the degree of ambivalence will moderate the relationship between leader-follower ambivalent relationships and individual outcomes. High degree of ambivalence means more incompatible leader-follower relationship. Specifically, the greater and incompatible the ambivalence, the easier to cause resource loss and get a positive outcome; and the smaller and complementary the ambivalence, the easier to acquire new resources. A high degree of ambivalence may occur when individuals hold opposing orientation on the same dimension (e.g., their feelings about leaders are simultaneously positive or negative, or they were undermined and supported simultaneously). A low degree of ambivalence may occur in the situation when the opposing parts seem contradictory but can exist simultaneously and work harmoniously. For example, leader narcissism and humility can exist simultaneously, as humility can alleviate the negative parts of narcissism, such as self-focus and overconfidence (Owens et al., 2015).

We suggest that high degree of ambivalence means irreconcilable opposing orientation, and this ambivalence makes followers feel more unpredictable of behavior of leader, more stressful, thus leading to a higher level of cognitive inflexibility (e.g., lower social self-esteem, lower self-efficacy), emotional inflexibility (e.g., negative affect), and behavioral inflexibility (e.g., rumination). To avoid these stressors and further resource loss, followers will adopt more disengagement behavior (e.g., avoid interaction with leaders), and all these inflexibility and disengagement behaviors will further have a deleterious effect on performance. Suurd Ralph (2019) found that within dimension ambivalence (perceive the leader as warmth and cold simultaneously or perceive the leader as competent and incompetent simultaneously) makes follower harder to understand, predict and influence leader compared with between dimension ambivalence (perceive leader as warmth but incompetent, or cold and competent).

*Proposition 1: The degree of ambivalence will moderate the relationship between leader-follower ambivalent relationship and outcomes; the higher the ambivalence, the more likely for the follower to have negative outcomes (more inflexibility, disengagement, and worse performance)*.

### Support From the Third Party

We argue that support from the third party will moderate the relationship between leader-follower ambivalent relationships and individual outcomes. That is, for followers with more support from third parties, they are more likely to get positive outcomes. Social support may come from different sources (e.g., coworker, organizations, friends, family members) and has different forms (e.g., instrumental support, emotional support). Social support is an important form of resources based on COR (Hobfoll, 1989).

Support from the third party is beneficial from two perspectives. First, support from third-party alleviates the detrimental effects of stress brought by leader–follower ambivalence. This argument is aligned with COR, which suggests that individuals with more resources are less vulnerable to resource loss (Hobfoll, 1989). Specifically, when followers are suffering from stressful ambivalence and negative affect, coworkers or family members would offer emotional support and some coping strategies that could lead to reductions in negative affect, physical strain, and perceived stress, and further even reduce the adverse attitude and behavior toward the leader. Social support also makes followers feel that they are cared for and loved (Uchino et al., 1996), which make them less suspicious of their worth. The stress-buffering effects of social support on physical flexibility (e.g., better cardiovascular regulation), emotional regulation (e.g. burnout, negative affect), and deviance work behavior have been widely recognized (Uchino et al., 1996; Halbesleben, 2006). Indeed, the moderating role of social support between stressor and negative outcomes, such as burnout, has been widely acknowledged (Etzion, 1984; Cohen and Wills, 1985; Hobman et al., 2009; Bliese et al., 2017).

Second, support from the third party is helpful for followers to interpret the ambivalence in a more integrative way and take advantage of the ambivalence. These arguments also echo with the COR theory, suggesting that individuals with more resources are more capable of resource gain (Hobfoll, 1989). Instrumental support from coworkers or workplace friendship may help followers to better interpret the ambivalent leadership by providing more information and diversified perspectives. Some empirical studies have demonstrated the beneficial effects of support from the third party. For example, leader support buffers the negative effects of the ambivalent relationship with a coworker (Duffy, 2002), and perceived organization support alleviate the negative influence of the ambivalent relationship with leaders (Lee et al., 2019).

*Proposition 2: Support from the third party will moderate the relationship between ambivalence and outcomes; the more support from third parties, the more likely for the followers to have positive outcomes (more flexibility, more engagement, and better performance)*.

### Follower's Integrative Complexity

We propose that integrative complexity of followers will moderate the relationship between leader–follower ambivalent relationships and individual outcomes. Integrative complexity was defined as “the capacity and willingness to acknowledge the legitimacy of competing perspectives on the same issue (differentiation) and to forge conceptual links among these perspectives (integration)” (Suedfeld et al., 1992). We expect that followers with higher integrative complexity are more likely to get positive outcomes. Followers with higher integrative complexity recognize and accept alternative perspectives and link different elements together, while followers with low integrative complexity prefer to focus on one-dimension rule when they interpret events or make decisions (Zhang et al., 2015).

We, thus, argued that integrative complexity of follower is both helpful in protecting resource loss and gaining new resources. For the former function, integrative complexity of follower itself can be regarded as a resource to help followers cope with the inconsistency and pressures brought by relationship ambivalence with leaders. Specifically, when faced with an ambivalent relationship with leaders, a follower with higher integrative complexity will try to interpret the ambivalence more validly and accept the ambivalence. Besides, they may change their opinion, which probably is the source of the ambivalence. By doing so, they suffered less stress and sense of unpredictability and fewer resources loss threats; thus, they are more likely to have emotional flexibility (positive affect), cognitive flexibility (openness to alternative explanation), and behavior flexibility (adaptive and proactive behavior), and they are less likely to involve in disengagement (such as avoid leader).

For acquiring new resources, followers with higher integrative complexity are more likely to interpret the ambivalence from different perspectives, integrate the ambivalence, and even find potential opportunities to learn from and make use of the ambivalence. For example, a follower with high integrative complexity is more likely to learn from emotional complexity of a leader, infer cognitive complexity of a leader, and predict what actions leaders may take. By observing and experiencing the ambivalence, followers transfer this possible uncertainty and threat (resources loss) to opportunity and resources (resources gain). It has been demonstrated that employees can make more effective decision when they are aware of the ambivalence (Guarana and Hernandez, 2016). In addition, scholars have suggested that paradox mindset (i.e., the willingness of individuals acceptance of tensions), which is similar to the meaning of integrative complexity, was found to alleviate the negative influences of tension (Miron-Spektor et al., 2018).

*Proposition 3: Integrative complexity of the follower will moderate the relationship between ambivalence and outcomes; the higher the integrative complexity of the follower, the more likely for them to have positive outcomes (more flexibility, more engagement, and better performance)*.

## Discussion

In this study, we review the definition of an ambivalent relationship, give a new definition to leader-follower ambivalent relationship in the workplace, and distinguish 10 different types of ambivalent leader-follower relationships based on the category of affect-based, cognition-based, complex-based, and behavior-based. From the reviewed studies, six different kinds of ambivalent leader-follower relationships were identified, and the behavior-related one attracts most attention. Several phenomena and terms were included in these ambivalent leader-follower relationship, such as paradoxical leadership, LMX ambivalence, and leader hypocrisy. In addition to these characteristics of ambivalent leader-follower relationship, it would be helpful for scholars to position these ambivalent leader-follower relationships in a wider leadership research. As we can see, these ambivalent leader-follower relationships can be categorized into three kinds based on their relationship with current leadership research. First, some of these ambivalent leader-follower relationships were defined based on previous leadership construct, such as LMX ambivalence, which was defined as co-existence of both positive and negative feelings toward leader-follower relationship (Lee et al., 2019). Second, some of the ambivalent leader-follower relationships occur because of the co-existence of different leadership behaviors, such as leader humility and narcissism (Owens et al., 2015; Zhang et al., 2017), and leader undermining and support (Duffy, 2002; Nahum-Shani et al., 2014). These first two categories supplement the current leadership construct and literature. Third, some other constructs were proposed by describing the ambivalent leader-follower relationships, such as paradoxical leadership (Zhang et al., 2015) and leader hypocrisy (Greenbaum et al., 2015). These constructs contribute to the leadership literature by introducing new concept, which deepens the understanding of leadership behaviors.

The ambivalent leader-follower relationships accompany both positive and negative outcomes. We further delineated the mechanism that explains these positive and negative outcomes, and under what conditions will ambivalent leader-follower relationships result in positive or negative outcomes. Specifically, we integrated COR to provide an integrative framework that explains the influences. These ambivalent leader-follower relationships can be regarded as resource loss because the ambivalence results in more workplace stressors (e.g., feelings of uncertainty and unpredictability). On the other hand, ambivalent leader-follower relationships can be regarded as resource gain because some forms of the ambivalence complement each other and bring positive outcomes. We, thus, proposed that the three moderators (degree of ambivalence, support from the third party, and integrative complexity of follower) may influence the outcomes to be positive or negative.

Current literature has illustrated several forms of ambivalent leader-follower relationships and their influences. The literature about these ambivalent relationships is still lacking compared with numerous leadership literature. To move this topic further, we discuss five further directions that are helpful for leader-follower ambivalent relationship research.

1. *More clarifications and other possible moderators on the influence of leader–follower ambivalent relationships are needed*. We propose that the three moderators would lead to possible or negative outcomes; while this is only an integrative framework, more studies are needed to deepen our understanding. For example, what determined the degree of ambivalence? Though we can measure the degree of ambivalence by asking perceptions of follower of the degree of ambivalence, it is helpful for us to know what makes leader–follower ambivalence in different degrees. The frequency, duration, types of ambivalence (e.g., affection or behavior), do they all matters? Is that possible that moderate ambivalence brings more positive outcomes? Because too much ambivalence makes the contradictory irreconcilable, while minor ambivalence may not lead to stress and also contribute limited new ideas or values. As for social support from the third party, we should distinguish a different support and find what is most effective. As indicated by previous study, the influence of work-related sources of social burnout is different from the non-work source of support (Halbesleben, 2006). Further, what is the relationship between these three moderators? For example, if a follower has extremely high integrative complexity, is it likely for him/her to transfer the extremely un-reconciled paradox into possible reconciled ones?
2. *The influence of leader–follower ambivalent relationships on leaders, teams, and organizations should be discussed the future*. In this study, we mainly focus on the influence of leader–follower ambivalent relationships on followers, which is also the focus based on current literature. What is the influence of ambivalent leader–follower relationships on leaders? Some scholars pointed out that leader inconsistent behavior results in distrust and condemnation (Effron et al., 2018). Is it possible that the ambivalent behavior of a leader is good for themselves? If the ambivalent leader–follower relationship is detrimental to leaders all the time, why do leaders involve in these ambivalent attitudes and behaviors, and which is regarded as deviant from the normal behavior? Furthermore, if these ambivalent relationships bring some beneficial effects, will it be the same case for the employees who show these forms of ambivalence? One possible explanation perspective is to regard the leader–follower interaction as a game from the game theory. It was demonstrated that the leaders will have advantage role in the interaction game, while if the leader acts like an average play, his/her payoff function value will be reduced (Nie and Zhang, 2008). We, thus, inferred that these ambivalent relationships are endorsement for leaders. In addition, the influence of ambivalent supervisor–follower relationships maybe different for different parties. For example, although workplace friendship was widely recognized to benefit individuals (Ingram and Roberts, 2000; Zou and Ingram, 2013; Methot et al., 2016), some other scholars argued the detrimental effect for organizations (Pillemer and Rothbard, 2018).
3. *The relationship between different types of ambivalent relationships, and the link between types and outcomes*. As we can see, although the leader–follower ambivalent relationship can be classified into 10 possible types, present research only focuses on six types. How about the other four types left? Some social network research covered other possible types in the workplace, for example, cognition (difficult to work tie) and behavior (seek task assistance tie) ambivalence is good for job performance (Brennecke, 2020). Does this conclusion also apply to leader–follower relationships? Besides, what is the influence and primacy of different types? Previous literature shows an individual would like to choose “loveable idiot” (choose the one they like while not so competent to interact), does that mean affection has more primacy than cognition? How about behavior, would behavior ambivalence be more powerful than other types? (Casciaro and Lobo, 2005, 2008, 2015). Third, the positives outcomes mainly come from ambivalent leader behavior, such as paradoxical leadership, visionary plus empowering leadership, and leader humility plus narcissism. Is that possible that other types are all detrimental? Although one exception now is that leader emotional complexity was argued to make followers have higher cognitive complexity; theoretically, we need further research to clarify the link between types and outcomes.
4. *Apply other possible theoretical mechanisms to understand the influence of ambivalent leader–follower relationships*. As we discussed earlier, the positive and negative influences of ambivalent leader–follower relationships can be interpreted as resource gain or loss. In addition to the COR perspectives, the positive and negative influences of ambivalent supervisor–follower relationship can also be regarded as job demands or resources from the job demand–resource model (JD-R, Demerouti et al., 2001). Specifically, the feelings of uncertainty, stress, and sense of unpredictability associated with ambivalent leader–follower relationships can be regarded as job demands. The complementary characteristics of ambivalent leader–follower relationship will result in better understanding and information, which can be regarded as job resources. Integrating JD-R models to ambivalent leader–follower relationship can help us to have more integrative view of ambivalent leader–follower relationships, and further explore and examine other possible influences of these ambivalent leader–follower relationships, such as burnout, engagement, commitment, absence, and well-being, which are the focus of JD-R (Bakker et al., 2003a,b).
5. *The dynamics and management of the ambivalent leader–follower relationship*. The ambivalent relationship is generally regarded as unstable, both would take some action to end, transfer this relationship (Ashforth et al., 2014). According to the opinion of Brennecke (2020), individuals get higher performance using ambivalent ties (seek task assistance from difficult to work ones), and the positive outcome mainly comes from the different resources and divergent thinking. In line with this argument, would leader–follower ambivalent relationships be more stable than others. because leaders always have more resources?

## Data Availability Statement

The original contributions presented in the study are included in the article/supplementary material, further inquiries can be directed to the corresponding authors.

## Author Contributions

In preparing this manuscript, WZ and QZ worked together to propose the research topic and developed the propositions. All authors contributed to the article and approved the submitted version.

## Conflict of Interest

The authors declare that the research was conducted in the absence of any commercial or financial relationships that could be construed as a potential conflict of interest.

## Publisher's Note

All claims expressed in this article are solely those of the authors and do not necessarily represent those of their affiliated organizations, or those of the publisher, the editors and the reviewers. Any product that may be evaluated in this article, or claim that may be made by its manufacturer, is not guaranteed or endorsed by the publisher.

## Footnotes

^1^We use this term from Yang et al. (2019), to describe negative ties that takes aspects of affective, behavioral, and cognitive facts and mixes them together to create multiplex, such as relationship conflict.

^2^The moderating effects of gender works because the different gender schema.
